# Supplementary material for: Low Zinc Alleviates the Progression of Thoracic Aortic Dissection by Inhibiting Inflammation
Source: Nutrients. 2023 Mar 28;15(7):1640. doi: 10.3390/nu15071640 (PMC10096567; doi:10.3390/nu15071640)
Supplement: Supplementary file 1 [file nutrients-15-01640-s001.zip › nutrients-2292163-supplementary.pdf]

Supplementary Table S1. Primers for Q-PCR analysis.

Gene	Forward	Reverse
β-Actin	CTATTGGCAACGAGCGGT	GGTCTTTACGGATGTCAACG
Col1A1	AGCAGACGGGAGTTTCTCCTC	GACATGTAGACTCTTTGCGGC
Cxcl2	GCGGTCAAAAAGTTTGCCTTG	CAGGTACGATCCAGGCTTCC
Fn1	ATGTGGACCCCTCCTGATAGT	GCCCAGTGATTTCAGCAAAGG
Myh11	CTCCCAACCTTTCCCTGACC	AGTGTGATTGCTCTGCTCCG
Acta2	GTTTTGTGGATCAGCGCCTC	TTCCTGACCACTAGAGGGGG
Myl9	GCGCCGAGGACTTTTCTTCT	TCTTGGCCTTGGCTCTCTTG
Cnn1	GCAGTGGACACACGCATTTT	AACAACTGGCCCCAAGACTC
Ramp1	GAGACTATTGGGAAGACGCTATG	CTCCTCCAGACCACCAGTG
TNF-α	CCCTCACACTCAGATCATCTTCT	GCTACGACGTGGGCTACAG
TNFAIP3	GAACAGCGATCAGGCCAGG	GGACAGTTGGGTGTCTCACATT
TNFSF10	ATGGTGATTTGCATAGTGCTCC	GCAAGCAGGGTCTGTTCAAGA
TNFSF9	CGGCGCTCCTCAGAGATAC	ATCCCGAACATTAACCGCAGG
TNFRSF1A	CCGGGAGAAGAGGGATAGCTT	TCGGACAGTCACTCACCAAGT
IL-1β	GCAACTGTTCCTGAACTCAACT	ATCTTTTGGGGTCCGTCAACT
IL-6	TAGTCCTTCCTACCCCAATTTCC	TTGGTCCTTAGCCACTCCTTC
IL-10	GCTCTTACTGACTGGCATGAG	CGCAGCTCTAGGAGCATGTG
MCP-1	TTAAAAACCTGGATCGGAACCAA	GCATTAGCTTCAGATTTACGGGT
MMP9	CTGGACAGCCAGACACTAAAG	CTCGCGGCAAGTCTTCAGAG
MMP2	CAAGTTCCCCGGCGATGTC	TTCTGGTCAAGGTCACCTGTC
ADAMTS	CATAACAATGCTGCTATGTGCG	TGTCCGGCTGCAACTTCAG

Supplementary Table S2. Antibodies for western blot.

Antibody	Vendor name	Catalog number	Dilution
β-Actin	Proteintech	81115-1-RR	1:5000
MYH11	Abcam	ab124679	1:1000
ACTA2	Cell signalling	19245	1:1000
CNN1	Proteintech	26689-1-AP	1:1000
TNF-α	Proteintech	60291-1-AP	1:1000
IL-1β	Proteintech	26048-1-AP	1:1000
IL-6	Abcam	ab259341	1:1000
